# Using Predictive Modeling Technique to Assess Core Temperature Adaptations from Heart Rate, Sweat Rate, and Thermal Sensation in Heat Acclimatization and Heat Acclimation

**DOI:** 10.3390/ijerph192013009

**Published:** 2022-10-11

**Authors:** Yasuki Sekiguchi, Courteney L. Benjamin, Ciara N. Manning, Cody R. Butler, Michael R. Szymanski, Erica M. Filep, Rebecca L. Stearns, Lindsay J. Distefano, Elaine C. Lee, Douglas J. Casa

**Affiliations:** 1 Korey Stringer Institute, Department of Kinesiology, University of Connecticut, Storrs, CT 06269, USA; 2 Sports Performance Laboratory, Department of Kinesiology and Sport Management, Texas Tech University, 3204 Main Street, Lubbock, TX 79409, USA; 3 Department of Kinesiology, Samford University, Birmingham, AL 35229, USA; 4 Department of Kinesiology and Military Science, Texas A&M University-Corpus Christi, Corpus Christi, TX 78412, USA; 5 Human Performance Laboratory, Department of Kinesiology, University of Connecticut, Storrs, CT 06269, USA

**Keywords:** heat acclimation, heat acclimatization, thermoregulation, exercise-heat stress, environmental exercise stress

## Abstract

Assessing the adaptation of rectal temperature (T_rec_) is critical following heat acclimatization (HAz) and heat acclimation (HA) because it is associated with exercise performance and safety; however, more feasible and valid methods need to be identified. The purpose of this study was to predict adaptations in T_rec_ from heart rate (HR), sweat rate (SR), and thermal sensation (TS) using predictive modeling techniques. Twenty-five male endurance athletes (age, 36 ± 12 y; VO_2max_, 57.5 ± 7.0 mL⋅kg^−1^⋅min^−1^) completed three trials consisting of 60 min running at 59.3 ± 1.7% vVO_2max_ in a hot environment. During trials, the highest HR and TS, SR, and T_rec_ at the end of trials were recorded. Following a baseline trial, participants performed HAz followed by a post-HAz trial and then completed five days HA, followed by a post-HA trial. A decision tree indicated cut-points of HR (<−13 bpm), SR (>0.3 L·h^−1^), and TS (≤−0.5) to predict lower T_rec_. When two or three variables met cut-points, the probability of accuracy of showing lower T_rec_ was 95.7%. Greater adaptations in T_rec_ were observed when two or three variables met cut-points (−0.71 ± 0.50 °C) compared to one (−0.13 ± 0.36 °C, *p* < 0.001) or zero (0.0 3 ± 0.38 °C, *p* < 0.001). Specificity was 0.96 when two or three variables met cut-points to predict lower T_rec_. These results suggest using heart rate, sweat rate, and thermal sensation adaptations to indicate that the adaptations in T_rec_ is beneficial following heat adaptations, especially in field settings, as a practical and noninvasive method.

## 1. Introduction

Heat acclimatization (HAz, training outdoors) and heat acclimation (HA, training in an artificial environment) are impactful strategies utilized to mitigate negative health and performance outcomes of exercise and environmental stress [1]. During HAz and HA induction, sufficient heat stress to elicit profuse sweating and elevated skin and internal body temperature is critical to induce positive physiological and performance adaptations [2]. The most rapid positive adaptations observed following HAz and HA are increased plasma volume and decreased heart rate (HR) typically occurring between 3 and 6 days [2,3]. Rating of perceived exertion (RPE) and thermal sensation (TS) also improve between 3 and 6 days [2,3]. Subsequent to multiple physiological mechanisms related to heat loss, one of the most important adaptations of HAz and HA is a decrease in internal body temperature at rest and during stressful exercise [2,4]. Lower internal body temperature during both rest and exercise allows for reduced fatigue at a given exercise intensity, which can lead to more effective training or enhanced performance [5]. Sweat rate (SR) increases traditionally between 10–14 days of HAz and HA [2]. The majority (75–80%) of adaptations resulting from HA occur within 4–7 days; however, these adaptations are dependent on the level of hyperthermia induced by heat exposure [2,6,7]. Although HAz and HA are methodologically different, adaptations observed following HAz and HA are typically similar, but much remains unknown about the differences in prescribing various forms of HAz and/or HA.

One challenge in studying the efficacy of HAz and HA protocols is that monitoring adaptations can require impractical or expensive and/or technically advanced methods. For example, an accurate measurement of internal body temperature requires rectal thermistors, esophageal thermistors, or ingestible pills [8]. Esophageal temperature is an invasive method and is not practical to use during exercise [9]. Even though rectal temperature (T_rec_) is widely used in laboratory settings, this method might not be practical or externally valid to use for continuous monitoring during exercise in field settings [9]. These measurements require connections between the thermistor and connecting devices, which is problematic for continuous monitoring [9]. Ingestible pills are relatively easy to use in a field setting; however, these pills may be cost-prohibitive and must also have some practical use limitations, such as ensuring the appropriate timing of ingestion for a valid measure. While there is an association between skin temperature and internal body temperature, skin temperature is affected by the surrounding environment and is not appropriate to use as “internal body temperature” [10]. Various devices that propose to accurately measure internal body temperature have limitations in sensitivity and specificity in detecting true health and safety risks associated with exercising in the heat. Assessing the adaptation of internal body temperature is critical following HAz and HA because it is associated with athlete performance and safety; however, more feasible and valid methods need to be identified [5,11].

HR, SR, and TS are relatively easier to collect and are reflective of physiological adaptation. The clear relationship between these variables and internal body temperature adaptations has not yet been quantitatively analyzed in a rigorous fashion and practical HAz and HA protocol. Thus, we aimed to 1: determine the relationship between HR, SR, TS, and T_rec_, and 2: determine if these variables can successfully predict changes in T_rec_ using predictive modeling techniques. Using two different induction protocols also allowed us to examine whether the predictive value of HR, SR, and TS applies to field-based and laboratory-based physiological outcomes of adaptations in the heat.

## 2. Materials and Methods

Male endurance trained athletes were recruited from the local community, including running through study flyers. Inclusion criteria were (1) VO_2max_ > 45 mL·kg^−1^·min^−1^, (2) 18–55 years old, (3) no history of heat illness and (4) no current injury limiting physical activity participation. Twenty-five male endurance athletes (mean (M) ± standard deviation (SD); age, 36 ± 12 y; height, 178.8 ± 6.4 cm; body mass, 73.0 ± 9.0 kg; VO_2max_ 57.5 ± 7.0 mL⋅kg^−1^⋅min^−1^; % body fat 10.7 ± 5.1%) participated in the study. Following an explanation of study procedures, which were approved by the Institutional Review Board (IRB) at The University of Connecticut, participants provided informed written consent to participate in this study. This study is a part of a large research study, and other research questions are answered in other manuscripts [12,13,14,15,16].

**Figure 1:**
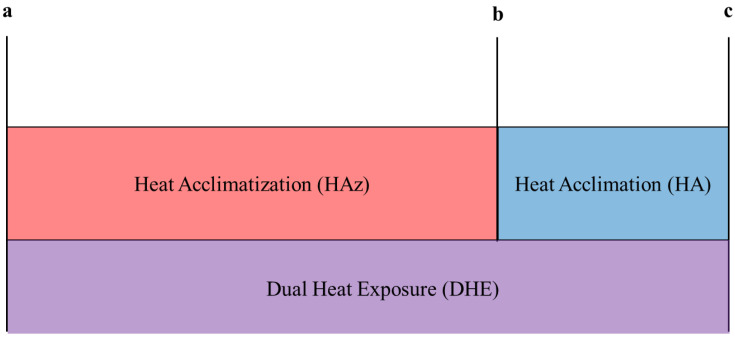
Self-directed summer training (HAz) and laboratory-based (HA) heat induction methods were used in sequence to assess the effects of each independent and together (DHE, HAz plus HA). Time points at which data were collected (**a**–**c**) represent different baseline, pre-, and post-values for isolating aspects of our protocol: (**a**) represents the baseline test for the entire study and represents pre-HAz and pre-DHE, (**b**) represents post-HAz and pre-HA, and (**c**) represents post-HA and post-DHE. Δ change for each induction method (HAz, HA, or DHE) was calculated as follows for the decision-tree analysis: (1) *HAz adaptations* = b − a, (2) *HA adaptations* = c − b, and (3) *DHE adaptations* = c − a.

Figure 1 demonstrated the study timeline. Participants performed three 60 min of steady-state exercise trials at 59.3 ± 1.7% vVO_2max_ on a treadmill (T150; COSMED, Traunstein, Germany) in an artificial environmental laboratory (M ± SD; ambient temperature (T_amb_), 35.3 ± 0.3 °C; relative humidity (%RH), 47.6 ± 0.6%; Wet Bulb Globe Temperature (WBGT), 29.3 ± 0.3 °C; wind speed, 4.0 ± 0.1 mph) at baseline, post-HAz, and post-HA (dual heat exposure). “Dual heat exposures” (DHE) is defined as HA following HAz. DHE allowed us to measure adaptations resulting from both HAz and HA together.

The baseline trial was performed when participants were unacclimatized. Upon arrival to the lab, participants provided urine samples to measure their hydration status before the 60 min exercise trials to confirm euhydration (M ± SD; urine specific gravity, 1.010 ± 0.009; urine color, 2 ± 1) [17]. Euhydration was defined as a USG < 1.020 and a urine color < 4 [17]. If the participant’s USG was ≥1.020 and ≤1.025, the participant consumed 500 mL of water before the trial. If the participant’s USG was >1.025, the trial was rescheduled for a different day. No fluid was provided throughout the 60 min exercise trial.

**Figure 2:**
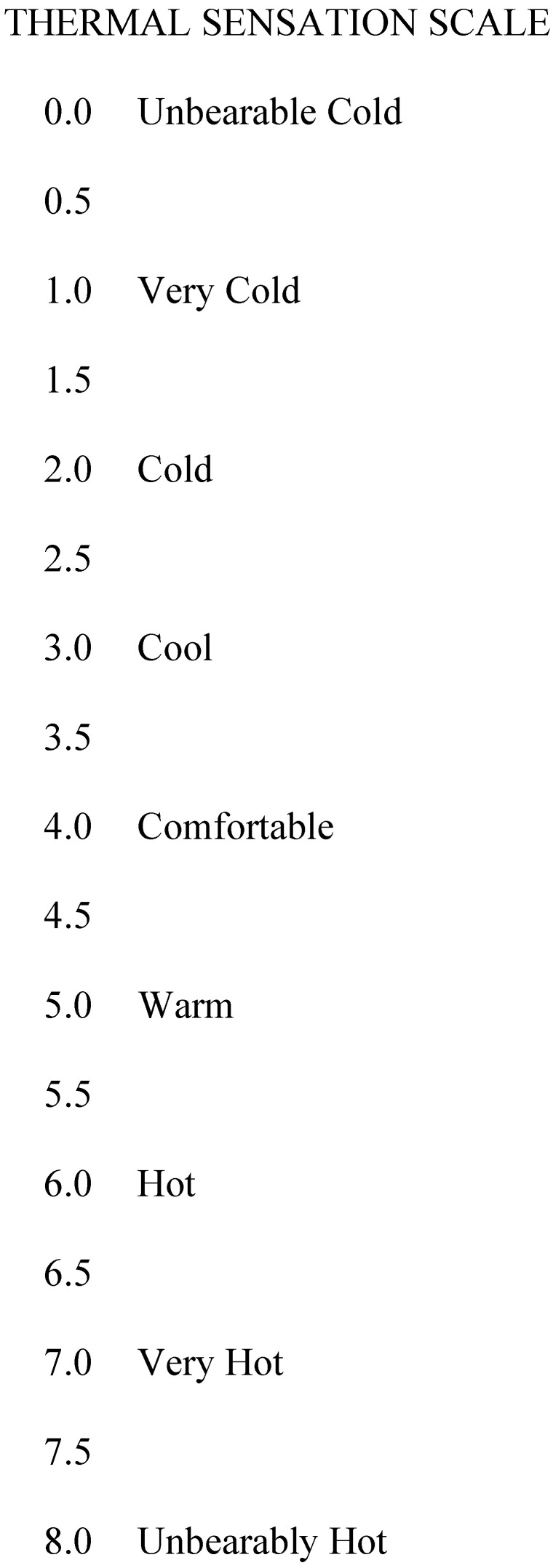
Thermal sensation scale [18].

During the trial, HR (H10^®^, Polar Electro™, Kempele, Finland), T_rec_ (MP160; BIOPAC Systems Inc., Goleta, CA, USA), and TS were recorded every five minutes. The TS was measured on a 0–8.0 Likert scale (0.0, Unbearable cold; 1.0, Very Cold; 2.0, Cold; 3.0, Cool; 4.0, Comfortable; 5.0, Warm; 6.0, Hot; 7.0, Very Hot; 8.0, Unbearably Hot; Figure 2) [18]. Nude body mass was collected pre- and post-trial to calculate SR (SR = pre-body mass − post-body mass − urine volume). In the current analysis, the highest HR and TS during the 60 min trial, T_rec_ at the end of the 60 min trial, and SR from the entire 60 min trial were used.

Following the baseline trial, participants performed self-directed summer training (HAz, 109 ± 9 days). In self-directed summer training, participants exercised outside using their normal training regimens. Training metrics, such as distance covered and HR, and environmental conditions for each training session were monitored. Detailed information was provided in other manuscripts [12,13]. After self-directed summer training, a post-HAz trial was performed to examine adaptations following HAz.

Then, participants completed a HA protocol, which consisted of five days of HA sessions over eight days in the heat (M ± SD; T_amb_, 39.1 ± 0.5 °C; %RH, 51.1 ± 2.3%; WBGT, 33.2 ± 0.8 °C). During the HA sessions, participants exercised to induce hyperthermia for 60 min, which is defined as “hyperthermic zone HA” (HZHA) method. Hyperthermia was defined as T_rec_ between 38.50 and 39.75 °C. Participants started sessions with a higher intensity exercise (~70% vVO_2max_) to increase T_rec_ rapidly to 38.5 °C and continued to exercise for the remaining 60 min with adjusted intensity to maintain T_rec_ in the hyperthermic zone. Following HA, a post-HA trial was performed to examine the adaptations.

The differences between (1) baseline trial and post-HAz trial (representative of *HAz adaptations*), (2) post-HAz trial (i.e., pre-HA trial) and post-HA trial (*HA adaptations*), and (3) baseline trial and post-HA trial (*DHE adaptations*) were calculated and entered into the decision tree analysis. Negative values for HR, TS, and T_rec_, and positive values for SR, indicated improvements (positive adaptations). In addition, T_rec_ for each participant and pre- and post-trial were categorized as either improved or not. Analyzing in a single analysis with Δ change for each induction method (HAz, HA, or DHE) standardizes the relative degree of relationship between predictive variables (HR, SR, TS) and the outcome (T_rec_) (Figure 1). This allows us to interpret whether predictive relationships hold independent of the induction method to determine cut-points.

A predictive modeling decision tree was used to determine the cut-points of HR, SR, and TS to indicate if T_rec_ was improved or not. The decision tree is a technique to classify outcomes by subdividing the set of observations into different subsets [19]. This process provides the most uniform subsets of observations possible [19]. The steps of the decision tree analysis include (1) setting the data to establish relationships between values of each variable (HR, SR, and TS) and if T_rec_ was improved or not, and (2) splitting values to minimize entropy with the variable [19]. After one split, the model provided the probability of improvement in T_rec_ when values for each variable (HR, SR, and TS) were higher or lower than the splitting values. This process was repeated to determine the best splitting values to use.

The best splitting values provided the highest probability and highest number of cases in which T_rec_ was improved. For example, when the splitting value was 5 bpm (e.g., HR at post-trial was lower than 5 bpm at pre-trial), the probability of T_rec_ improvements was 78% with 41 cases of observations. However, when the splitting value was 13 bpm, the probability of T_rec_ improvements was 82% with 22 cases. The cut-point for each variable was determined based on the balance between probability and the number of observations to avoid overfitting the model and to achieve the best overall probability when three variables were used together, which is explained below.

After the cut-points were determined, binary logistic regressions were used to predict changes in T_rec_ from one variable. Then, the number of variables among HR, SR, and TS to meet the cut-points was counted. One-way ANOVA with LSD was performed to examine the difference of T_rec_ between the number of variables meeting cut-points. Additionally, positive and negative likelihood ratios were calculated, and a ROC analysis was performed to determine the sensitivity and specificity for each number of variables that met cut-points. Data are reported as M ± SD, 95% confidence intervals (CI) and effect size (ES). ES was calculated using Hedges’ g with the effects identified as either small (0.2–0.49), medium (0.5–0.79), or large (>0.8) effects [20]. Statistical significance was set to *p* ≤ 0.05, a priori. Statistical analyses and predictive modeling analyses were completed using SPSS Statistics, version 25 (IBM Corp., Armonk, NY, USA) and JMP, version 15 (SAS Institute Inc., Caryn, NC, USA).

## 3. Results

The average training duration at each HAz session was 58.3 ± 76.5 min for running and 94.5 ± 70.8 min for cycling. Average HR was 139 ± 14 bpm for running and 128 ± 16 bpm for cycling during HAz. The average WBGT at each HAz session was 22.2 ± 4.3 °C for running and 23.7 ± 4.0 °C for cycling. Duration, average T_rec_, average T_rec_ for hyperthermia period, average HR, and average HR for hyperthermia period during heat acclimation from Day 1 to 5 are presented in Table 1.

Average HR, T_rec_, and TS were significantly lower at post-HA (HR, 134 ± 11 bpm; T_rec_, 38.03 ± 0.39 °C; TS 5.1 ± 0.6) compared to both baseline (HR, 143 ± 12 bpm, *p* < 0.001; T_rec_, 38.29 ± 0.37 °C, *p* = 0.005; TS, 5.6 ± 0.6, *p* = 0.001) and post-HAz (HR, 138 ± 14 bpm, *p* = 0.013; T_rec_, 38.25 ± 0.42 °C, *p* = 0.009; TS, 5.5 ± 0.5, *p* = 0.003). In addition, HR was significantly lower at post-HAz (*p* = 0.002) compared to baseline. The sweat rate was significantly higher post-HA (1.93 ± 0.46 L⋅h^−1^) compared to post-HAz (1.76 ± 0.43 L⋅h^−1^, *p* = 0.027). Detailed data and analyses during HAz, and HA as well as at baseline, post-HAz, and post-HA trials are provided in another manuscript [12,13].

The cut-point of HR was determined to be when HR in post-trial was 13 bpm lower than pre-trial (HR < −13 bpm). This cut-point indicated the probability of 81.8% accuracy of improved T_rec_. Logistic regression demonstrated that using the HR cut-point by itself was not a significant predictor of T_rec_ improvements (*p* = 0.064). The cut-point of SR was determined to be an SR greater than 0.3 L·h^−1^ (sweat rate > 0.3 L·h^−1^) post-trial compared to pre-trial. This cut-point showed the probability of 88.2% accuracy. Logistic regression indicated that this cut-point significantly predicted T_rec_ improvements; however, the r^2^ was low (r^2^ = 0.07, *p* = 0.022). The cut-point of TS was a TS lower than 0.5 (TS ≤ −0.5) post-trial compared to pre-trial. This cut-point indicated the probability of 77.8% accuracy. Logistic regression indicated that this cut-point significantly predicted T_rec_ improvements; however, the r^2^ was also low (r^2^ = 0.08, *p* = 0.013).

When two or three of the HR, SR, and TS variables met the identified cut-points, T_rec_ was improved for 22 out of 23 cases, which coincided with a probability of 95.7% accuracy. When only one variable met the cut-point, T_rec_ was improved for 22 out of 36 cases, with a probability of accuracy of 61.1%. When no variable met the cut-point, T_rec_ was improved in 6 out of 16 cases, with a probability of accuracy of 37.5%.

**Figure 3:**
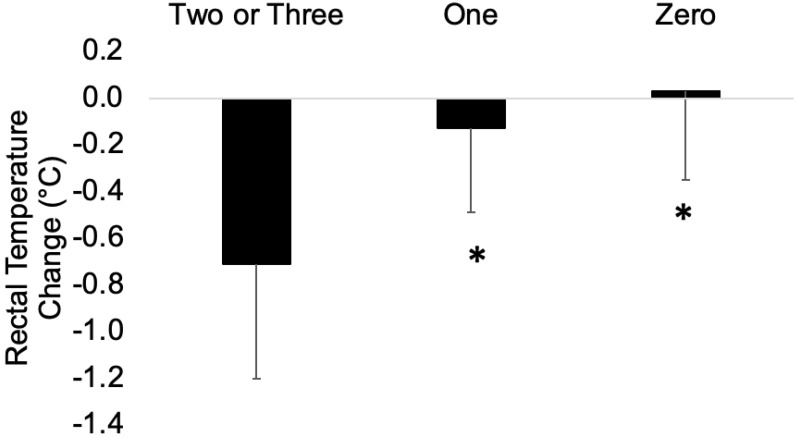
The differences in rectal temperature between pre- and post-test when two or three variables, one, and zero variables met the cut-points. * indicates statistical significance from when two or three variables met the cut-points, *p* ≤ 0.05.

The differences of T_rec_ between pre- and post-trials when two or three variables met the cut-points (−0.71 ± 0.49 °C) indicated significantly greater improvement compared to when one (M ± SD [95% CI]; −0.13 ± 0.36 °C [−0.80, −0.37], ES = 1.42, *p* < 0.001) or no variables (0.03 ± 0.38 °C [−1.01, −0.48], ES = 1.67, *p* < 0.001) met the cut-point with large effects (Figure 3). No differences were found in T_rec_ between pre- and post-trials when less than one variable met the cut-points (*p* = 0.207).

The positive likelihood ratio was 11.0, and the negative likelihood ratio was 0.58 when two or three variables met the cut-points. However, the positive likelihood ratio was 2.2, and the negative likelihood ratio was 0.2 when more than one variable met the cut-points. Additionally, specificity was 0.96 when two or three variables met cut-points and sensitivity was 0.44. However, specificity was 0.60 and sensitivity was 0.88 when more than one variable met cut-points.

## 4. Discussion

The purpose of this study was to determine the relationship between HR, SR, TS, and T_rec_ and if these variables can successfully predict changes in T_rec_ by using predictive modeling techniques. When two or three variables met the cut-points (HR, <−13 bpm; SR, >0.3 L·h^−1^; TS, ≤−0.5), T_rec_ was improved 96% of the time with a specificity of 0.96. In addition, the level of adaptations in T_rec_ was significantly greater when two or three variables met the cut-points compared to one or zero variables with large effects. These results provide sports scientists, coaches, and medical professionals with a practical method to monitor adaptations in T_rec_ following HAz and HA, especially in field settings.

**Figure 4:**
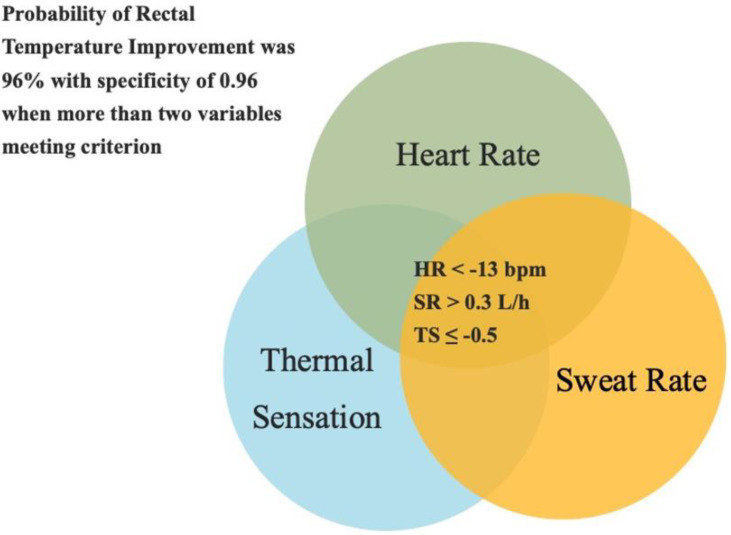
A Venn diagram consisting of heart rate, thermal sensation, and sweat rate to indicate if rectal temperature adaptation occurred or not. Cut-points were heat rate < −13 bpm, sweat rate > 0.3 L·h^−1^, and thermal sensation ≤ −0.5 between pre- and post-trials. When three or two variables met cut-points, the rectal temperature decreased in 96% of the cases.

HR, SR, and TS are easy metrics to measure during exercise even in field settings. HR measures only require HR straps, SR is calculated through body mass loss, and TS is determined using a perceptual scale. While assessing internal body temperature through T_rec_ is a common method used in laboratory settings, it might not be practical to utilize for continuous monitoring during exercise in field settings [9]. It is critical to monitor adaptations of internal body temperature to ensure improvements in athlete safety and exercise performance. Monitoring only one variable such as HR, SR, or TS only provides a 78% to 88% accuracy in indicating improvements in T_rec_. Although this level of accuracy was found, there was no statistical significance for HR in determining improvements in T_rec_. SR and TS alone, although statistically significant, had an r^2^ value of 0.07 and 0.08, indicating that this variable alone can only explain 7% and 8% of the improvements in T_rec_. However, measuring three variables together achieved 96% accuracy with a specificity of 0.96 in determining lower T_rec_. This shows that measuring all three of these variables is necessary to accurately determine improvements in T_rec_. A Venn diagram consisting of HR, SR, and TS is a useful tool to assume the adaptation in T_rec_ (Figure 4). This method allows for monitoring adaptations following HAz, HA, and DHE (HAz plus HA) in a practical, time-efficient, and cost-effective manner.

The reasons that HR, SR, and TS were included as predictive variables is because they are time- and cost-efficient with good practicality in field settings. Adaptations in these variables are typically observed over different time courses [2,3,21]. For example, adaptations in HR and TS are normally observed earlier than an adaptation in T_rec_ [2]. However, an adaptation in SR is one of the last adaptions to be induced [2]. The different time courses in which these adaptations occur supports the theory that these factors individually cannot accurately predict improvements in T_rec_. Responses of HR, SR, TS, and T_rec_ to exercise in the heat are related to each other, and these variables represent both cardiovascular and thermoregulatory systems [22,23]. Thus, these measurements can collectively provide comprehensive information related to adaptations following HAz and HA.

This study suggests using the cut-points for HR < −13 bpm, SR > 0.3 L·h^−1^, and TS ≤ −0.5 in endurance-trained athletes with a wide range of ages (19–55 years old) is effective in assessing adaptation in T_rec_. These cut-points can be used to assess adaptations regardless of induction methods, such as HAz, HA, and DHE (HAz plus HA). However, the cut-points to predict an adaptation in T_rec_ may differ among less aerobically fit populations. There are no thresholds for HR, SR, TS, and T_rec_ to indicate “enough”, “successful”, or “optimal” adaptations following HAz and HA. Thus, the decision tree was used to determine the cut-points for HR, SR, and TS in the current study. The cut-point used demonstrated that the magnitude of improvement in T_rec_ at the end of the trial was 0.58 °C greater and 0.74 °C greater when two or three variables met the cut-points compared to one variable and zero variables, respectively. Both indicated large effects. The highest HR and TS, SR, and T_rec_ at the end of the trial were collected from 60 min of exercise at 60% vVO_2max_, which was performed T_amb_ at 35.3 ± 0.3 °C, %RH at 47.6 ± 0.6%, WBGT at 29.3 ± 0.3 °C. This exercise intensity corresponded to an average of 77% HR_max_ for each individual. This procedure can be used to assess the adaptations following HAz and HA. However, different environmental conditions, exercise intensities, and durations of exercise during trial procedures can provide different results for adaptations seen with each variable.

## 5. Conclusions

A Venn Diagram consisting of the HR, SR, and TS with cut-points of HR < −13 bpm, SR > 0.3 L·h^−1^, and TS < −0.5 between pre- and post-trial was a useful tool to assess the adaptations in T_rec_. When two or three variables met cut-points, the probability of accuracy to indicate lower T_rec_ was 96% with a specificity of 0.96. When one and zero variables met the cut-point, the probability of accuracy was 61% and 38%. Additionally, the magnitude of adaptations was significantly greater when two or three variables met compared to one or zero. These results suggest using heart rate, sweat rate, and thermal sensation adaptations to indicate that the adaptations in T_rec_ are beneficial following heat adaptations, especially in field settings, as a practical and noninvasive method.

## Figures and Tables

**Table 1:** Duration, average rectal temperature (T_rec_), average T_rec_ for hyperthermia period, average heart rate (HR), and average HR for hyperthermia period during heat acclimation from Day 1 to 5.

	Day 1	Day 2	Day 3	Day 4	Day 5
Duration (min)	82.0 ± 6.3	81.0 ± 6.0	84.6 ± 5.6	83.4 ± 8.3	83.0 ± 8.2
Ave T_rec_ (°C)	38.85 ± 0.42	38.93 ± 0.31	38.81 ± 0.38	38.80 ± 0.30	38.78 ± 0.31
Ave T_rec_ for hyperthermia (°C)	39.16 ± 0.42	39.24 ± 0.22	39.16 ± 0.36	39.16 ± 0.30	39.11 ± 0.22
Ave HR (bpm)	137 ± 13	132 ± 14	132 ± 11	131 ± 12	129 ± 12
Ave HR for hyperthermia (bpm)	138 ± 14	132 ± 14	131 ± 14	131 ± 14	128 ± 12

## Data Availability

Data are not available as other manuscripts are still in the writing process.
